# Plasminogen activator inhibitor-1 4G/5G gene polymorphism and primary open-angle glaucoma

**Published:** 2008-07-04

**Authors:** Georg Mossböck, Martin Weger, Christoph Faschinger, Otto Schmut, Wilfried Renner

**Affiliations:** 1 Department of Ophthalmology, Medical University of Graz, Graz, Austria; 2 Clinical Institute of Medical and Chemical Laboratory Diagnostics, Medical University of Graz, Graz, Austria

## Abstract

**Purpose:**

Alterations of the plasmin system have been suggested to participate in the multifactorial pathogenesis of primary open-angle glaucoma (POAG). The main physiological inhibitor of the plasmin system is plasminogen activator inhibitor-1 (*PAI-1*), which leads to decreased degradation of extracellular material. Interestingly, elevated PAI-1 levels in the aqueous humor of patients with POAG have been reported. A common polymorphism within the promoter region (*PAI-1 4G/5G*) has previously been shown to reduce the gene transcription rate of *PAI-1*. The purpose of the present study was to investigate a hypothesized association between *PAI-1 4G/5G* and the presence of POAG in a Caucasian population.

**Methods:**

The present case-control study comprised 212 unrelated patients with POAG and 212 healthy control subjects, matched for age and sex. Genotyping of *PAI-1 4G/5G* polymorphisms was done using polymerase chain reaction.

**Results:**

Allelic frequencies and genotype distributions of *PAI-1 4G/5G* did not significantly differ between patients with POAG and control subjects (*PAI-1 4G/5G*: 29.7% versus 29.7%). Presence of the *PAI-1 4G*-allele was associated with a nonsignificant odds ratio of 0.98 (95% confidence interval: 0.74–1.30) for POAG.

**Conclusions:**

Our data suggest that *PAI-1 4G/5G* itself is unlikely to be a major risk factor among Caucasian patients with POAG.

## Introduction

Primary open-angle glaucoma (POAG; OMIM 137760) is one of the major causes of blindness throughout the world [1]. Elevated intraocular pressure (IOP) is the most important and so far the only therapeutically modifiable risk factor [2]. Accumulation of extracellular material (ECM) in the trabecular meshwork, which may be due to enhanced production or reduced degradation of ECM, has been suggested to lead to elevated IOP [3,4].

The serine protease plasmin has a central position in the fibrinolytic pathway, thereby restoring blood flow after thrombotic events. In addition, it also has a key role in the degradation and proteolysis of ECM via modulation of growth factors, cytokines, and metalloproteinases [5]. Plasminogen is converted to plasmin by the action of urokinase (u-PA) and tissue plasminogen activator (t-PA). The main physiological inhibitor of u-PA and t-PA is plasminogen activator inhibitor-1 (PAI-1; OMIM 173360), a glycoprotein composed of 379 amino acids, which forms a stable complex with both u-PA and t-PA [6]. It was shown that PAI-1 protects ECM from t-PA and plasminogen mediated degradation [7].

There is ample evidence for a physiological role of the plasmin system in the anterior segment of the eye and the trabecular outflow system in particular. Activity of u-PA, t-PA, and PAI-1 has been found in the human aqueous humor [8-11]. In an in vitro study, Shuman and coworkers [12] demonstrated that in trabecular meshwork cells, t-PA activity outweighs the inhibitor activity. Fuchshofer and coworkers [13] found a significant increase of PAI-1 expression in cultures of human TM cells after treatment with TGF-β2, which leads to the expression of active matrix metalloproteinase-2 (MMP-2). Reduction of resistance to aqueous outflow was observed after perfusion of monkey eyes with plasmin [14]. It can thus be hypothesized that increased levels of *PAI-1* may lead to decreased proteolysis of ECM in the trabecular meshwork, subsequently leading to an increase in IOP. Indeed, two recent studies reported elevated PAI-1 levels in the aqueous humor of patients with open-angle glaucoma compared to cataract patients [15,16].

A common functional polymorphism within the promoter region due to a single guanosine insertion/deletion, 4G/5G (rs1799889), has been identified [17]. The *PAI-1 5G* allele leads to binding of a repressor protein thereby decreasing gene transcription. Consequently, higher plasma PAI-1 concentrations have been found among homozygotes for the 4G- allele compared to heterozygotes and homozygotes for the 5G-allele [17-19]. However, a recent study found no association between the *PAI-1 4G/5G* polymorphism and expression of PAI-1 in platelets [20].

Since POAG is a disease with a high heritability as shown in population based studies [21,22], our study was set to investigate a hypothesized association between the *PAI-1* 4G/5G polymorphism and the presence of POAG.

## Methods

The present study was comprised of 212 patients with POAG and 212 control subjects. All participants were seen at the Department of Ophthalmology, Medical University Graz (Graz, Autria) between September 2002 and November 2005. All participants were Caucasians from the same geographical area in the southern part of Austria. Informed consent was obtained from all subjects before enrollment. The study was conducted in accordance with the standards of the local ethics committee and the National Gene Technology Act.

All patients underwent slit lamp biomicroscopy as well as testing for best corrected visual acuity, Goldmann applanation tonometry, gonioscopy, pachymetry, and standard automated perimetry (Interzeag Octopus 101, program G2) or – in cases of profoundly decreased visual acuity – Goldmann perimetry. In all patients, photographs of the optic disc were taken. POAG was defined by an intraocular pressure of at least 21 mmHg before the initiation of a pressure-lowering therapy, an open anterior chamber angle, optic disc changes characteristic for glaucoma (increased vertical cup/disc ratio of more than or equal to 0.7), visual field defects characteristic for glaucoma (inferior or superior arcuate scotoma, nasal step, or paracentral scotoma), and absence of conditions leading to secondary glaucoma. Optic discs were assessed by glaucoma specialists (GM and CF).

The control group consisted of 212 patients with no morphological or functional damage indicative for primary or secondary open-angle or angle closure glaucoma. Control subjects were admitted to our department for cataract surgery and were matched to cases by sex and age (±3 years). Medical history concerning arterial hypertension and diabetes mellitus was obtained from all participants.

### Genotype determination

Genomic DNA was isolated from venous blood by standard methods and stored at –20 °C. *PAI-1* genotypes were determined by a 5′-exonuclease assay (TaqMan, Applied Biosystems, Vienna, Austria). Primer and probe sets were designed and manufactured using Applied Biosystems ‘Assay-by-Design’ custom service (Applera, Austria). The polymerase chain reaction (PCR) was performed in a Primus 96 plus thermal cycler (MWG Biotech AG, Ebersberg, Germany) using a total volume of 5 μl containing 2.5 μl Universal Genotyping MasterMix (Applied Biosystems), 0.125 μl 40X Assay-by-Design mix (Applied Biosystems), 0.375 μl H_2_O, and 2 μl DNA. Reactions were overlaid with 15 μl of mineral oil. Cycling parameters were as follows primary denaturation was for 10 min at 94 °C followed by 40 cycles for 20 s at 92 °C and for 1 min at 60 °C. Fluorescence was measured in a lambda Fluoro 320 Plus plate reader (MWG Biotech AG) using excitation/emission filters of 485 nm/530 nm for FAM-labeled probes (4G-allele) and 530 nm/572 nm for VIC-labeled probes (5G-allele). The data were exported into Excel format and depicted and analyzed as a scatter plot.

To validate the genotyping method, we also determined *PAI-1* genotypes of 24 samples, which had been analyzed previously by a polymerase chain reaction and endonuclease digestion method [23]. Results of both methods were consistent in all samples.

### Statistical analysis

Descriptive statistics were used to calculate frequencies and percentages of discrete variables. Continuous data are given as mean±standard deviation (SD). Means were compared using the Mann–Whitney test. Proportions of groups were compared by χ^2^ test. Odds ratio (OR) and 95% confidence interval (95% CI) were calculated by logistic regression. The criterion for statistical significance was p≤0.05. Statistical analysis was done using the SPSS statistical package (SPSS, version 14.0, Chicago, Illinois).

## Results

The present study comprised 212 patients (125 female and 87 male) with POAG and 212 controls (125 female and 87 male). The mean age of patients was 71.5±9.8 years, and the mean age of control subjects was 70.9±9.6 years. Clinical characteristics of patients and control subjects are shown in Table 1. Patients had a mean deviation of 11.5±6.7 dB, a mean loss of variance of 38.0±27.6 square decibel, a mean intraocular pressure of 21.8±15.4 mmHg, and a mean cup disc ratio of 0.79±0.13 in the worse eye. In 34 patients (16%), a trabeculectomy with mitomycin C has been performed, and in 19 patients (9%), a transscleral cyclophotocoagulation has been performed while 44 patients (20.8%) had both interventions. Seventy-two patients (34%) were treated with prostaglandin analogs, 44 patients (20.8%) were treated with β-blockers, 23 patients (10.8%) were treated with brimonidine, and 17 patients (8%) were treated with carbonic anhydrase inhibitors.

**Table 1:** Clinical characteristics of patients with primary open-angle glaucoma and controls.

**Characteristics**	**Patients with POAG (n=212)**	**Control subjects (n=212)**	**Significance p value**
Mean age (±SD)	71.5±9.8	70.9±9.6	0.32
Range (years)	39.5–88.3	40.8–89.8	
Female*	125 (59.0)	125 (59.0)	
Arterial hypertension*	127 (59.9)	116 (54.7)	0.28
Diabetes mellitus*	36 (17.0)	39 (18.4)	0.7

*Numbers are given as n (%).

No significant differences in either genotype distribution or allelic frequencies of the *PAI-1 4G/5G* polymorphism were found between patients with POAG and control subjects (Table 2). Presence of the *PAI-1 4G*-allele was associated with an odds ratio of 0.98 (95% CI: 0.74–1.30) for POAG. The present study had a statistical power of 0.80 to detect an odds ratio of 1.77 for the *PAI-1 4G/5G* genotype in patients with POAG.

**Table 2:** Genotype distribution and allele frequency of *PAI-1* 4G/5G polymorphism.

**Polymorphism**	**Patients with POAG (n=212)**	**Control subjects (n=212)**	**Significance p value**
PAI-1 4G/4G*	63 (29.7)	63 (29.7)	1
4G/5G*	111 (52.4)	113 (53.3)	0.84
5G/5G*	38 (17.9)	36 (17.0)	0.79
*PAI-1* 4G-allele frequency	0.559	0.563	0.96

*Numbers for genotypes are n (%)

The observed genotype distributions did not deviate from those predicted by the Hardy-Weinberg equilibrium, and for control subjects, the genotype distributions were similar to those reported for Caucasian populations [24,25].

## Discussion

The balance between plasmin activators and inhibitors is of utmost importance not only for vascular homeostasis but also for extracellular proteolysis. Alterations of the plasmin system potentially leading to reduced degradation of ECM in the trabecular meshwork have been implicated in the pathogenesis of POAG. Interestingly, elevated levels of PAI-1 in the aqueous humor of patients with POAG were reported only recently [15,16]. Gene polymorphisms leading to increased synthesis of PAI-1 may thus contribute to the pathogenesis of POAG. To the best of our knowledge, the present study is the first to investigate a hypothesized association between *PAI-1 4G/5G* and the presence of POAG.

Genotypes of *PAI-1 4G/5G* were determined in 212 patients with POAG and 212 control subjects that were matched for age and sex. Allelic frequencies as well as genotype distributions did not significantly differ between both groups. As the study has a statistical power of 0.80 to detect an odds ratio of 1.77 for the *PAI-1 4G/5G* genotype in patients with POAG, our data suggest that the investigated polymorphism is unlikely a major genetic risk factor for POAG in Caucasian patients.

Potentially, elevation of the PAI-1 level in the aqueous humor may result from reduced aqueous humor turnover in patients with POAG. Furthermore, synthesis of PAI-1 is regulated by various factors. Hyperglycemia as well as insulin and insulin precursor molecules stimulate the transcription of *PAI-1* [26-28]. Angiotensin II, very low density lipoproteins, and unsaturated fatty acids induce the expression of PAI-1 whereas estrogen suppresses synthesis of PAI-1 [29-32]. Moreover, cytokines like TGF-β2 and tumor necrosis factor-α increase PAI-1 expression [33-35]. In an in vitro study, Fleenor and coworkers provided evidence that treatment of trabecular meshwork with TGF-β2 increased secretion of PAI-1, which led to elevated IOP [33]. Thus, our finding that the *PAI-1* 4G/5G polymorphism is not associated with an increased risk for POAG does not exclude a substantial role of PAI-1 in the pathogenesis of POAG.

In conclusion, no statistically significant difference in the genotype distribution of the *PAI-1 4G/5G* polymorphism was found between patients with POAG and control subjects, which strongly suggests that this polymorphism itself is unlikely a major risk-factor for POAG.
